# Prediction and attenuation of seasonal spillover of parasites between wild and domestic ungulates in an arid mixed‐use system

**DOI:** 10.1111/1365-2664.13083

**Published:** 2018-01-28

**Authors:** Josephine G. Walker, Kate E. Evans, Hannah Rose Vineer, Jan A. van Wyk, Eric R. Morgan

**Affiliations:** ^1^ School of Biological Sciences University of Bristol Bristol UK; ^2^ Cabot Institute University of Bristol Bristol UK; ^3^ Elephants for Africa Maun Botswana; ^4^ Population Health Sciences Bristol Medical School University of Bristol Bristol UK; ^5^ School of Veterinary Sciences University of Bristol Bristol UK; ^6^ Department of Veterinary Tropical Diseases Faculty of Veterinary Science University of Pretoria Onderstepoort South Africa; ^7^ Institute for Global Food Security Queen's University Belfast Belfast UK

**Keywords:** basic reproduction quotient, generalist, helminths, host switching, parasites, *Q*_0_, reservoir host, ruminants, ungulates

## Abstract

1. Transmission of parasites between host species affects host population dynamics, interspecific competition, and ecosystem structure and function. In areas where wild and domestic herbivores share grazing land, management of parasites in livestock may affect or be affected by sympatric wildlife due to cross‐species transmission.
2. We develop a novel method for simulating transmission potential based on both biotic and abiotic factors in a semi‐arid system in Botswana. Optimal timing of antiparasitic treatment in livestock is then compared under a variety of alternative host scenarios, including seasonally migrating wild hosts.
3. In this region, rainfall is the primary driver of seasonality of transmission, but wildlife migration leads to spatial differences in the effectiveness of treatment in domestic animals. Additionally, competent migratory wildlife hosts move parasites across the landscape.
4. Simulated transmission potential matches observed patterns of clinical disease in livestock in the study area. Increased wildlife contact is correlated with a decrease in disease, suggesting that non‐competent wild hosts may attenuate transmission by removing infective parasite larvae from livestock pasture.
5. Optimising the timing of treatment according to within‐year rainfall patterns was considerably more effective than treating at a standard time of year. By targeting treatment in this way, efficient control can be achieved, mitigating parasite spillover from wildlife where it does occur.
6. *Synthesis and applications*. This model of parasite transmission potential enables evidence‐based management of parasite spillover between wild and domestic species in a spatio‐temporally dynamic system. It can be applied in other mixed‐use systems to mitigate parasite transmission under altered climate scenarios or changes in host ranges.

## Abstract 2

This model of parasite transmission potential enables evidence‐based management of parasite spillover between wild and domestic species in a spatio‐temporally dynamic system. It can be applied in other mixed‐use systems to mitigate parasite transmission under altered climate scenarios or changes in host ranges.

## 1 INTRODUCTION

Disease transmission between domestic and wild animals can have important impacts on agricultural economics (Alexandersen, Zhang, & Donaldson, 2002; Renwick, White, & Bengis, 2007) and conservation (Smith, Acevedo‐Whitehouse, & Pedersen, 2009). Gastrointestinal nematodes (worms) are a major determinant of host health, production and fitness in wild ungulate as well as domestic populations (Gulland, 1992, 1995; Perry & Randolph, 1999). Worm transmission varies in time and space, and this variation is driven by complex biotic and abiotic factors. Rainfall, temperature and pasture characteristics determine development and survival of free‐living stages, while host density, behaviour and diversity influence the chances of free‐living stages entering a suitable host (Ezenwa, 2003, 2004; Fox, Marion, Davidson, White, & Hutchings, 2013; Morgan, Milner‐Gulland, Torgerson, & Medley, 2004). Where livestock share grazing land with wildlife, such as at the border of conservation areas, many parasite species infect both wild and domestic hosts (Walker & Morgan, 2014; Walker, Plein, Morgan, & Vesk, 2017), and human management of parasites in livestock has the potential to affect the parasites in wildlife through spillover between host species (Weinstein & Lafferty, 2015). Parasite transmission studies nevertheless tend to focus on single‐host, single‐parasite systems, due to the inherent complexity and often empirical intractability of an integrated ecological approach (Buhnerkempe, Roberts, Dobson, Heesterbeek, Hudson, & Lloyd‐Smith, 2015; Walker & Morgan, 2014). Predictive disease models offer a way to identify the risks of such transmission and to design effective interventions, and their use is growing (Cowled, Garner, Negus, & Ward, 2012; McCallum, 2016; Morgan et al., 2006).

Standard practice for managing helminth parasites in livestock is through anthelmintic treatment, but this is inefficient and has led to globally widespread anthelmintic resistance in both intensive and extensive livestock systems (Mahieu, Ferré, Madassamy, & Mandonnet, 2014; Van Wyk, 2001). More sustainable management is needed (Charlier et al., 2014). The characteristics of an optimal management strategy that is also sustainable and robust will depend on the ecological context of the livestock production system in question.

We focus on a case study of the Makgadikgadi and Nxai Pans National Park (MPNP) region of Botswana, a semi‐arid region with highly seasonal rainfall. Humans, domestic animals and wildlife coexist and share limited resources, and there are high degrees of overlap between domestic and wild ungulates. In arid and semi‐arid ecosystems such as this one, rainfall limits the survival and development of free‐living stages of worms, while also driving seasonal changes in potential host diversity due to migration or the concentration of grazers around seasonally scarce resources.

The focal parasite is the well‐studied generalist nematode *Haemonchus contortus*, although the approach could be extended to other worms. This parasite infects at least six species of wild and domestic ungulate in the study area (Walker et al., 2017) and has been demonstrated to affect the health of livestock locally (Walker et al., 2015). *Haemonchus contortus* is highly fecund, and development of infectious stages outside the host is linked closely to climate, leading to strong seasonal fluctuations in infection due to rapid population growth when conditions are favourable.

In this study, we evaluate the potential for parasite transmission between host species in the MPNP region by measuring overlap between wild and domestic host species and parasite burden in goats. To understand the drivers of transmission, we use a novel adaptation of an ecological and epidemiological model (Rose, Wang, Van Dijk, & Morgan, 2015), which uses abiotic factors (temperature and rainfall) to predict seasonal patterns in parasite development and risk of transmission. We apply this model to optimise treatment timing in livestock in the face of spatial and seasonal variation in climate and host overlap. Finally, by incorporating biotic and abiotic factors, we predict the direction and magnitude of parasite spillover between species. Our overall aim is to describe specific and local applications to improve parasite management in an ecological context in the case study, while also developing methodologies that are more broadly applicable to parasite management in support of conservation and agriculture in other systems.

## 2 MATERIALS AND METHODS

### 2.1 Spatial and seasonal variability in shared grazing

**Figure 1:**
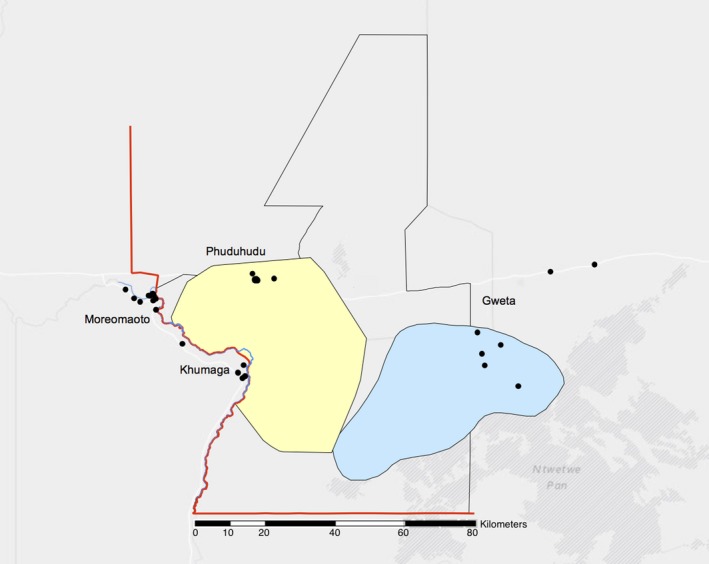
A map of the study area showing the locations of the four study villages and enrolled kraals (black dots) which make up each village, and range of zebra migration (wet season: blue, dry season: yellow) in the Makgadikgadi and Nxai Pans National Park (MPNP) region. Note the fence (red line), river (blue line) and park boundary (black line) overlap on the west side of the park. Adapted from Bradley (2012)

The study area consisted of the MPNP in northeast‐central Botswana and four villages which border the park (Walker et al., 2015; Figure 1). Livestock production in this region is conducted by smallholders and animals graze on communal pastures. Livestock, including cattle, goats, sheep, donkeys and horses, are released in the morning to graze freely and are housed overnight in kraals (rough‐fenced enclosures). The area is semi‐arid, and rainfall is highly seasonal: around 450 mm of rain falls mainly between November and March (Department of Environmental Affairs & Centre for Applied Research, 2010). Livestock kept in the villages vary in levels of overlap in grazing sites with wildlife as a result of varying distance from the park, barriers such as the river and park border fence, and seasonal migration of wild ungulates (Figure 1). Wild ungulates, mainly wildebeest and zebra, migrate seasonally, spending the dry season in the west near the river and the wet season grazing ephemeral grasses in the east. We used questionnaires to assess the extent of shared grazing between wild and domestic animals in the four villages (Walker et al., 2015). To confirm the seasonal presence of migratory wildlife, road surveys were conducted on the west side of the park in which all observed wild mammalian herbivore species were recorded. These surveys were carried out once a month from September 2013 through August 2014 (except for November, December and June) on a set route along established unsurfaced roads inside the national park in three separate transect lines totalling 69.1 km. The route was driven at 20 km/hr with researchers looking at both sides of the road; transect direction alternated each month.

### 2.2 Spatial and seasonal variability in parasite burden in goats

Goats are a key resource in the study area, with 39%–45% of households owning goats for reasons including investment and for food (Statistics Botswana, 2014; Walker et al., 2015). They were therefore chosen as the focal livestock species. We examined goats in the study villages for clinical signs of infection with worms in October 2013 (prior to the rainy season), and again in March–April 2014 (after the rainy season, during which time some animals were treated; Walker et al., 2015). We assessed FAMACHA score as a measure of anaemia caused by *H. contortus* infection (Bath & Van Wyk, 2009) and assigned scores to anaemic (≥3) or non‐anaemic (*<*3) categories. The FAMACHA system allows farmers to estimate ocular mucous membrane colour as an indication of anaemia, which is most often caused by haemonchosis (Malan, Van Wyk, & Wessels, 2001).

The effect of village, location (east/west) and presence or absence of a fence (Figure 1) on anaemia before and after the rainy season were assessed by logistic regression, using the glm function in r version 3.1.0 (R Core Team, 2014). Goat characteristics at enrolment were covariates: age, sex, girth and—in the post‐rainy season analysis—whether the herd was treated. We selected the best model for each of the two time points by Akaike information criterion (AIC), comparing with the null model using the likelihood ratio test.

### 2.3 Mechanistic (*Q*_0_) model of seasonal patterns in parasite development

To determine the impact of abiotic factors on transmission, we simulated the potential for *H. contortus* eggs deposited throughout the year to develop successfully and infect new hosts, using the basic reproduction quotient, *Q*_0_. For helminths, *Q*_0_ is defined as the expected number of offspring that reach reproductive maturity produced by one adult worm during its lifetime, in the absence of density‐dependent constraints (Heesterbeek & Roberts, 1995). *Q*_0_ is similar to the basic reproduction ratio *R*_0_ of microparasites, but projects potential parasite population growth and not multiplication of infected hosts, and also encapsulates time‐varying transition rates between parasite life stages (Roberts & Heesterbeek, 1995). Density‐dependent constraints such as host immunity are intentionally omitted from *Q*_0_, which rather seeks to identify instantaneous conditions under which transmission is most favoured. Among helminths, *H. contortus* life history lends itself well to the *Q*_0_ framework, as egg production per female worm is high (~5,000 eggs per day) and development to infective larvae either fails or occurs quickly (Saccareau et al., 2017). Therefore, current external conditions for transmission are a useful predictor of population trajectory, even if population magnitude is further modified by variations in susceptibility and egg output.

Our simulation adapted the GLOWORM‐FL model (Rose et al., 2015), which predicts worm population dynamics from daily rainfall, evaporation and mean temperature. These climatic inputs were gathered on a daily time‐scale from the Africa Drought Monitor, which uses satellite data and land surface models to estimate climate variables (Sheffield et al., 2014). The models were run using climate data from July 2003 to November 2015 for locations on the east and west side of the study area, with parameters specific to *H. contortus* drawn from Rose et al. (2015).

The GLOWORM‐FL model (Rose et al., 2015) tracks the development of the free‐living stages of worms from eggs (*E* and newly deposited eggs *E*_new_) through larval stages in faeces *L*_12_ and *L*_3f_, with mortality (μ), development (δ), initial survival (*C*) and larval movement (*m*) parameters that are functions of daily temperature and moisture.

(1) $$\frac{dE}{dt}\;=\;-\left(\mu_{1}\left(t\right)\;+\;2\delta \left(t\right)\right)E\;+\;E_{\mathrm{new}}C\left(t\right)$$

(2) $$\frac{dL_{12}}{dt}\;=\;-\left(\mu_{2}\left(t\right)\;+\;2\delta \left(t\right)\right)L_{12}\;+\;2\delta \left(t\right)E$$

(3) $$\frac{dL_{3f}}{dt}\;=\;-\left(\mu_{3}\left(t\right)\;+\;m_{1}\left(t\right)\right)L_{3f}\;+\;2\delta \left(t\right)L_{12}$$

We extended the model to include two hosts which consume *L*_3_ from the herbage and become infected with adult female worms,

(4) $$\begin{array}{cc} \frac{dL_{3p}}{dt}= & -\mu_{4}\left(t\right)L_{3p}\left(1-m_{2}\left(t\right)\right)-\left(\mu_{5}\left(t\right)+\frac{\beta \rho_{s}+\beta \rho_{v}}{\gamma (t)}\right)L_{3p}m_{2}\left(t\right)\;\;\;\\ & +\;L_{3f}m_{1}\left(t\right) \end{array}$$

(5) $$\frac{dH_{i}}{dt}=m_{2}\left(t\right)L_{3p}\left(\frac{\beta \rho_{i}}{\gamma (t)}\right)\epsilon_{i}\omega_{i}$$

where *L*_3p_ represents *L*_3_ on pasture (in the soil or on herbage); μ is the substrate‐specific (faeces, soil or herbage) mortality rate of larvae; *m*_1_ is the horizontal migration rate of *L*_3_ out of faeces (*L*_3f_) and onto pasture (*L*_3p_); *m*_2_ is the proportion of *L*_3p_ on herbage; β is the herbage consumed per day; ρ_*i*_ is relative density where *i* represents either the stationary host *s* or migratory host *v*; and γ(t) is the herbage density at a given time point *t*. Parameters are listed in Table 1. The primary host *s* represents non‐migratory goats, and the alternative host *v* represents either a migratory (wild) or stationary (wild or domestic) host depending on the modelled scenario (Table 2). The number of adult female worms in the hosts, *H*_*i*_, is determined by the *L*_3_ consumed from the pasture, and ϵ is the proportion of those consumed that establish as adults (i.e., are not killed by innate immunity), which is effectively a function of host species susceptibility. ω is the proportion of female worms. Satellite‐derived NDVI was used in the model to account for the change in herbage density between the wet and dry seasons (Sheffield et al., 2014) as described in Appendix S1.

**Table 1:** Parameter definitions, and functions or ranges used in the model, including those from Rose et al. (2015). Parameters that vary with time depend on *T* = mean daily temperature, *P* = daily precipitation, *E* = daily potential evapotranspiration or *V* = NDVI, of which all input values from Sheffield et al. (2014)

Parameter	Definition	Values	Source
δ	Development rate from egg to *L*_3_	−0.09746 + 0.01063*T*	Rose et al. (2015)
μ_1_(*t*)	Egg mortality rate	exp(−1.62026 − 0.17771*T* + 0.00629*T* ^2^)	Rose et al. (2015)
μ_2_(*t*)	*L*_1_ and *L*_2_ mortality rate	exp(−1.82300 − 0.14180*T* + 0.00405*T* ^2^)	Rose et al. (2015)
μ_3_(*t*)	*L*_3_ mortality rate in faeces	exp(−2.63080 − 0.14407*T* + 0.00463*T* ^2^)	Rose et al. (2015)
μ_4_(*t*)	*L*_3_ mortality rate in soil	exp(−3.68423 − 0.25346*T* + 0.00740*T* ^2^)	Rose et al. (2015)
μ_5_(*t*)	*L*_3_ mortality rate on herbage	Same as μ_3_(*t*)	Rose et al. (2015)
*m*_1_(*t*)	Horizontal migration (translation) of *L*_3_ onto pasture	$\left\{\begin{array}{cc} 0.25, & P\geq 2\\ 0, & P<2\;\text{and}\sum_{i=-4}^{t}P_{i}/E_{i}<1\\ 0.051, & P<2\;\text{and}\sum_{i=-4}^{t}P_{i}/E_{i}\geq 1 \end{array}\right.$	Rose et al. (2015)
*m*_2_(*t*)	Proportion of total pasture *L*_3_ on herbage	exp(−5.48240 + 0.45329*T* − 0.01252*T* ^2^)	Rose et al. (2015)
*C*(*t*)	Development success correction factor	$\left\{\begin{array}{cc} 0.1, & \sum_{i=4}^{t}P_{i}/E_{i}<1\\ 0, & \sum_{i=4}^{t}P_{i}/E_{i}\geq 1 \end{array}\right.$	Rose et al. (2015)
β	Herbage consumed per day	1	
ρ	Host density^a^	0, 0.1, 1, or 10	Brooks and Maude (2010), Chief Wildlife Officer (2012)
γ(*t*)	Herbage density scaling factor	1 or V	
ϵ	Establishment rate^a^	0, 0.05, 0.25, 0.5, or 1	Barger and Le Jambre (1988); Jacquiet et al. (1998)
ω	Proportion of female worms	0.5	Fleming (1988)
*l*	Development time of infective larvae to egg‐producing adult worms in the host	14 days	Anderson (2000)
*f*	Life span of adult worm in the host	55 days (14‐100)	Barger and Le Jambre (1988); Kao et al. (2000)

a Host density and establishment rate differ for each host scenario, and each combination of these parameter values was simulated according to the ranges in (Table 2).

**Table 2:** Alternative host scenarios modelled. In each, the primary host is assumed to be small ruminants (sheep and goats), at density = 1. Relative density of the second host is based on ranges of recently reported densities in Central and Ngamiland Districts, Nxai Pans National Park and Countrywide in Botswana (Chief Wildlife Officer, 2012). Competence is expected competence of Host 2 relative to Host 1 for *Haemonchus contortus*. Competence of Host 1 is assumed to be 0.5

Second host	Establishment (ϵ range)	Density (ρ ratio range)	Migration
Cattle	Low (0.05–0.5)	2–5*x* (1–10)	No
Impala	Unknown (0.05–1)	0.05–1*x* (0.01–1)	No
Zebra	Zero (0)	0.05–1*x* (0.01–1)	Yes
Wildebeest	Unknown (0.05–1)	0.02*x* (0.01–1)	Yes

First, to identify contrasting peaks in *L*_3_ availability in different areas as a result of abiotic factors, independent of host density or immunity, we simulated the number of *L*_3_ on pasture over time assuming constant egg output from a single adult female worm on either the east or west side of the park, and no secondary hosts. For comparison, we ran two alternative models, holding either temperature or rainfall constant at the overall mean value for the predicted period, to determine the relative effect of variation in these factors on *L*_3_ availability compared to the standard model.

Next, to calculate the value of *Q*_0_ for day τ, a female worm was assumed to reach sexual maturity in a goat host on that day, and the total number of successful female offspring of the initial worm was projected as follows. The initial worm is assumed to live and produce 5,000 eggs daily for *f* days, such that *Q*_0_ at time τ is the sum of the female adult worms that establish in both hosts from the offspring of the initial worm,

(6) $$Q_{0\tau }=\sum_{j=\tau }^{\tau +f}q_{\mathrm{sj}}+q_{\mathrm{vj}}$$

where the total number of female adult worms that establish in each host *i *= *s* or *i *= *v* from the 5,000 daily deposited eggs was calculated as *q*_*i*τ_ = max (*H*_*i*_) . *Hi* was calculated from the model in Equations (1), (2), (3), (4), (5) for each day τ from 1 July 2003 through 30 June 2014 by simulating the deposition of *E*_new_ **= 5,000 on day τ and projecting the development of these eggs until τ + 365.

The model was run in r version 3.1.1 (R Core Team, 2014) using the differential equation solver package deSolve (Soetaert, Petzoldt, & Setzer, 2010). Model ability at predicting timing of clinical cases was assessed as described in Appendix S1.

### 2.4 Optimal treatment timing

Treatment was simulated by eliminating daily egg output for a time period representing the residual effect of a given anthelmintic. This was calculated first in the absence of hosts by scaling the reduction in *L*_3_ on pasture from treatment on a given day to the total *L*_3_ on pasture in the year of treatment and the following year, and second including hosts, where the reduction in total *Q*_0_ for treatment on each day τ was calculated as

(7) $$Q_{0\tau }^{*}=\sum_{j=\tau }^{\tau +z}q_{\mathrm{sj}}+q_{\mathrm{vj}}$$

where *z* is the length of time the treatment is effective. In both cases, the impact of treatment was calculated for two anthelmintics that are widely used in Botswana (see Appendix S1).

The most effective treatment timing in terms of *L*_3_ reduction on pasture was compared between years (1 July to 30 June such that rainy seasons were not split between years). Correlation of reduction in *L*_3_ on pasture with daily and cumulative rainfall measures was assessed in order to suggest the best time to treat during the rainy season.

To evaluate differences in treatment effectiveness under host scenarios, the reduction in *Q*_0_ due to treatment within the baseline model in which there is only one available host was compared to a set of alternative scenarios of wildlife presence (Table 2). Four scenarios represent realistic alternative hosts (cattle, zebra, wildebeest and impala) based on the results of the field surveys described above, with different densities relative to the primary domestic host (assuming random mixing) and various levels of parasite establishment (ϵ). Migration was modelled as being triggered either by month or by weekly precipitation (see Appendix S1).

### 2.5 Migratory hosts and parasite movement

To assess the role of migration in moving worms between the east and west of the study area, we assumed the presence of a second host. There are many potential characteristics of a secondary host; for simplicity, we assume the migratory host has the same density and establishment rate as the primary host. This allows for a simple baseline scenario from which to calculate the impact of migration, in that without migration the secondary host would contribute to 50% of the worm burden on pasture. We then calculated how the contribution to worm burden differs if the secondary host migrates. We estimated the worm burden *B* on each day *d* in the stationary and migratory hosts *H*_*i*_ in each location *y* (east or west). The source location was specified as *x* (local or distant to *y*), and the model again assumed constant egg deposition in each location to represent a single generation of worms,

(8) $$B_{\mathrm{ixyd}}=\sum_{t=d-l-f}^{d-l}H_{\mathrm{ixyt}}$$

where *t* is a daily time step, *l* is a lag in days to account for development from larvae to adult in the host, and *f* is the adult worm life span in days (Table 1). For each host and location (east or west), we then calculated the proportion of the total burden during 2003–2014 attributable to each source location.

## 3 RESULTS

### 3.1 Spatial and seasonal variability in shared grazing

Farmers reported that wild and domestic ungulates use the same spaces. As expected, wildlife were more commonly reported to enter villages not fenced off from the park (Phuduhudu and Gweta). Livestock from those villages also entered the park (Table 3), although farmers in Khumaga (fenced) also reported moderate levels of wildlife contact. Respondents reported shared water and pasture use by wild and domestic species (Table S1 in Appendix S2). Villages close to the river (Khumaga and Moreomaoto) reported higher overlap in water use compared to pasture use, while Gweta and Phuduhudu reported higher overlap in pasture use than water use.

**Table 3:** Percentage of farmers reporting wildlife species entering the village, and livestock entering the park, by village (questionnaire sample size)

	Gweta (8)	Khumaga (11)	Moreomaoto (11)	Phuduhudu (33)
African Elephant	100	100	100	97
Giraffe	0	27	0	88
Hippopotamus	13	45	18	6
Impala	38	27	9	61
Blue Wildebeest	63	45	9	94
Plains Zebra	88	55	9	97
Livestock enter park	63	45	9	100

**Figure 2:**
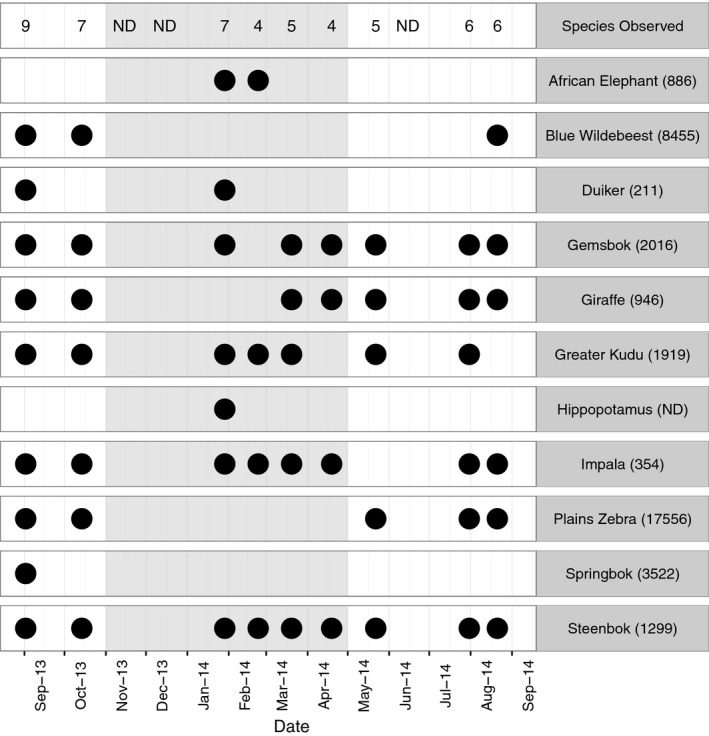
Results of road surveys on west side of park, and in brackets, the mean abundance of wildlife species from seven aerial surveys of Makgadikgadi and Nxai Pans National Park (MPNP) between 1996 and 2006, ND = no data (Brooks & Maude, 2010). Black circles indicate the species was observed during the road survey in a given month, and grey shading denotes the rainy season. Species Observed (top row of figure) shows total number of species observed in each road survey, with ND indicating that no surveys were conducted in November, December or June

The road survey of wildlife on the western side of the national park confirmed the seasonality of the abundant migratory species zebra and wildebeest, which were observed in this area only in the dry season; other ungulates were observed throughout the year (Figure 2).

### 3.2 Spatial and seasonal variability in parasite burden in goats

The relationship between anaemia and village‐level covariates was different in dry and rainy seasons (Table 4). In the dry season, goat‐level covariates age, sex and girth all contributed to a better fit model (AIC 1016). Adding village led to the best fit model (AIC 997, likelihood ratio test χ^2^ = 24.7 on 3 *df*,* p* < .0001); in this model, a goat in Khumaga was less than half as likely to be anaemic as a goat in Gweta, while the other villages were not significantly different from Gweta. Including fence instead of village slightly improved the fit of the base model (AIC 1014, χ^2^ = 4.15 on 1 *df*,* p *=* *.042), with the presence of the fence associated with lower levels of anaemia. Village location (east/west) was not significantly different from the base model (AIC 1018, χ^2^ = 0.087 on 1 *df*,* p *=* *.77).

**Table 4:** Logistic regression results for location category associated with anaemia (clinical sign of haemonchosis, measured by FAMACHA score) in the wet and dry seasons. Village (pairwise results shown), fence and location are separate models, selected by Akaike information criterion (AIC) as described in the text. The dependent variable is coded as non‐anaemic = 0, anaemic = 1, *n* = number of goats. Intercept and covariates goat age, sex, girth and treatment not shown, but are included in all models (see text). For ease of interpretation, villages separated from the park by a fence are indicated in italics, while the eastern village (Gweta) is shown in bold text

Dependent variable (*n*)	Category	Reference	*B*	*SE*	Wald *z*	Odds ratio (95% CI)
Dry season anaemia (964)	*Khumaga*	**Gweta**	−0.902	0.263	−3.43	0.406 (0.239–0.672)**
	*Khumaga*	*Moreomaoto*	−1.215	0.282	−4.31	0.297 (0.169–0.511)***
	*Khumaga*	Phuduhudu	−1.096	0.262	−4.18	0.334 (0.197–0.552)***
	*Moreomaoto*	**Gweta**	0.313	0.228	1.37	1.367 (0.873–2.137)
	Phuduhudu	**Gweta**	0.194	0.208	0.93	1.214 (0.808–1.827)
	Phuduhudu	*Moreomaoto*	−0.119	0.227	−0.524	0.888 (0.569–1.388)
	Fence	No fence	−0.336	0.166	−2.026	0.714 (0.515–0.987)*
Rainy season anaemia (678)	*Khumaga*	**Gweta**	1.749	0.440	3.972	5.751 (2.453–13.872)***
	*Khumaga*	*Moreomaoto*	−0.413	0.307	−1.348	0.661 (0.361–1.203)
	*Khumaga*	Phuduhudu	0.753	0.378	1.993	2.123 (1.019–4.499)*
	*Moreomaoto*	**Gweta**	2.163	0.369	5.857	8.694 (4.289–18.360)***
	Phuduhudu	**Gweta**	0.996	0.380	2.624	2.708 (1.299–5.805)**
	Phuduhudu	*Moreomaoto*	−1.166	0.314	−3.710	0.312 (0.166–0.572)**
	Fence	No Fence	1.499	0.279	5.368	4.477 (2.598–7.784)***
	East	West	−1.549	0.339	−4.571	0.212 (0.106–0.404)***

Significance indicated by *p < .05, **p < .01, ***p < .0001.

After the rainy season, goat age and whether the herd was treated contributed to a better fit model (AIC 615); inclusion of girth and sex did not change AIC but were included for consistency. Model fit was improved by including fence (AIC 588), village (582) or location (592): all models were significantly different from the base model by the likelihood ratio test (*p < *.0001). At this time of year, the presence of the fence was associated with higher levels of anaemia, and villages in the east of the range with lower levels of anaemia.

### 3.3 Seasonal patterns in parasite development

**Figure 3:**
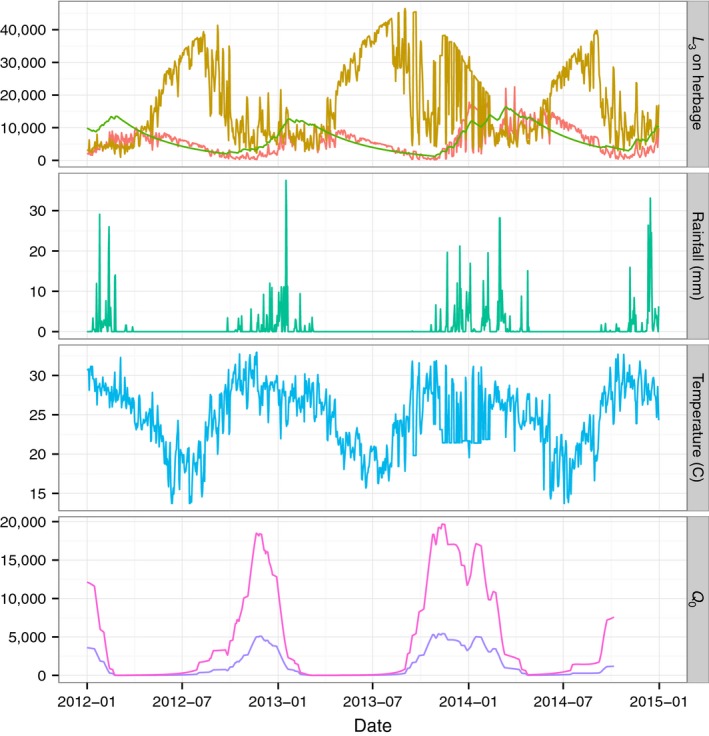
Predicted *L*_3_ on pasture for 2012–2015 using climate data from Gweta (east of the park). Top panel shows three models, standard model (red line), constant temperature (green line), constant precipitation (brown line). Middle panels show climate data used in the standard model: rainfall (green line); temperature (blue line); bottom panel, *Q*_0_ for the same time period, with (purple line) and without (pink line) seasonal grass growth (NDVI)

Seasonality of larval development on pasture was primarily driven in the model by rainfall, although temperature also played a role (Figure 3). When rainfall was held constant in the simulation, the pattern of *L*_3_ survival changed in both quantity and timing. When temperature was held constant, the timing of the peaks changed slightly, but the overall pattern was similar. Interannual variation in temperature was low, while interannual variation in precipitation was high, driving differences in the timing and quantity of *L*_3_ that develop on pasture. Including NDVI to represent seasonal grass growth did not affect timing, but did affect the magnitude of *Q*_0_, such that the rainy season peaks were lower as increased vegetation diluted available larvae (Figure 3, bottom panel).

The best correlation between observed cases of anaemia and model output was found with predicted *Q*_0_ with no time lag; *Q*_0_ was a better predictor than season or precipitation (Table S2 in Appendix S2). Due to autocorrelation, the correlation between *Q*_0_ and clinical cases is cyclical, with inverse correlation at lag 6 weeks or greater. *Q*_0_ calculated with NDVI led to a better model than without, but in all models the variance explained by the fixed effect was only about 1%.

### 3.4 Optimal treatment timing

The greatest reduction in *L*_3_ on pasture was achieved in the model by treating during a period of rainfall. Very little reduction in *L*_3_ was achieved by treatment during the dry season (normally April through September). However, the optimal timing of treatment within the rainy season varied from year to year (Figure S1 in Appendix S2), and the change in *L*_3_ due to treatment was correlated with daily precipitation (for the 14‐day treatment, Spearman's ρ = 0.45, *p < *.0001; for the 35‐day treatment, Spearman's ρ = 0.42, *p < *.0001).

If farmers were to treat on the optimal day from the previous year, the total reduction in *L*_3_ on pasture over the simulated period would be approximately 36% as effective as if treatment were given on the optimal day for the current year. By treating each year on the mean most effective date from the whole simulated period (28 January), the total effect on *L*_3_ would be approximately 38% as effective as treating on the optimal treatment date.

**Figure 4:**
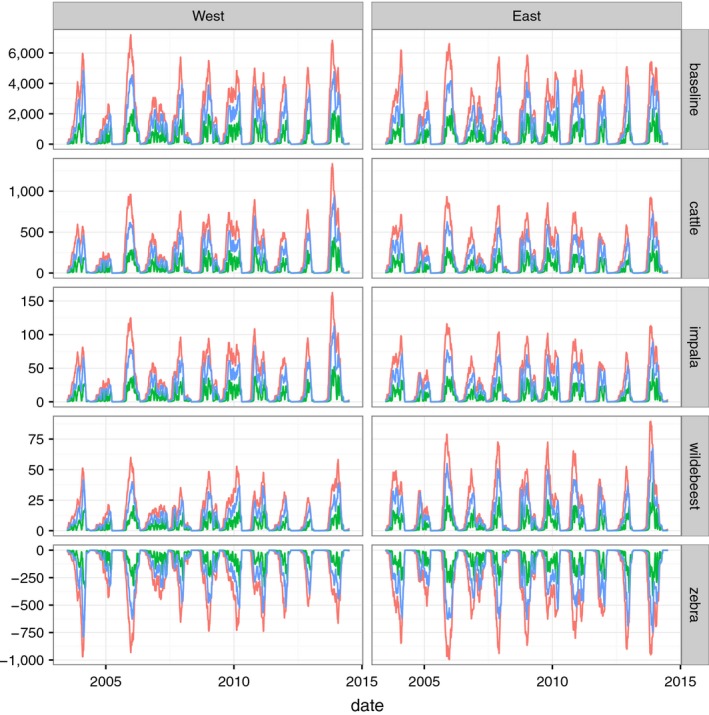
Total *Q*_0_ (red line) and reduction in *Q*_0_ due to 35‐day treatment (blue line) or 14‐day treatment (green line) under realistic alternative host scenarios representing shared grazing between goats and cattle, impala, zebra or wildebeest. The top row shows the baseline scenario in which there is only one host, and below rows show the difference in *Q*_0_ and treatment impact compared to the baseline. Columns show the results for the east and west of the study area. An intermediate scenario for each alternative host (Table 2) is shown as an example to demonstrate differences due to relative density (ρ), establishment (ϵ) and migration (month‐based migration scenario for wildebeest and zebra): baseline, ρ = 1*,* ϵ = 0.5; cattle, ρ = 1, ϵ = 0.25; impala (non‐migratory) and wildebeest (migratory), ρ = 0.1*,* ϵ = 0.25; zebra, ρ = 1*,* ϵ = 0. Note different *y*‐axis scales showing differences from baseline values

As with *L*_3_ on pasture, the date of optimal treatment in the presence of alternative hosts showed high variation between years, but was similar between host scenarios. Competent alternative hosts slightly increased total *Q*_0_, while non‐competent hosts slightly decreased it (Figure 4). In the baseline scenario, total *Q*_0_ was higher in the east in some years and in the west in others; this reflects slightly different rainfall patterns in the two locations. For both *Q*_0_ and the treatment effect in the alternative host scenarios, the range in the parameter values of ρ (density) and ϵ (establishment) affected the height of the peaks but not their timing (Figure S2 in Appendix S2).

### 3.5 Migratory hosts and parasite movement

Migrating secondary hosts contributed less than stationary secondary hosts to the total worm burden in each location (Table S3 in Appendix S2), but when migratory hosts were present, a small proportion of the worm burden in each location was sometimes transferred from the other location by the migratory hosts (see Appendix S2).

## 4 DISCUSSION

In this study, we evaluated the extent of shared grazing between wild and domestic species and applied a novel modelling approach to examine parasite transmission in a complex, multihost system. The model simulated the seasonality of nematode transmission to goats and was used to explore the likely magnitude and timing of parasite transmission between goats and sympatric wild ungulate populations. Further, it identified optimal times of anthelmintic treatment of goats to attenuate onward transmission. This approach offers a way of integrating ecological factors into parasite control in livestock kept in mixed‐use areas, which should support farmer livelihoods and decrease conflict due to disease, thereby promoting co‐existence.

The timing of antiparasitic treatment in goats was optimised by maximising the reduction in onward transmission from a single treatment. Transmission was highly correlated with the seasonal pattern of rainfall, and so the best results followed treatment during the rainy season. The optimal treatment date within the rainy season, however, was sensitive to the high variability in rainfall from year to year. The same patterns occured in the presence of alternative hosts. A strategy that aims to reduce transmission should therefore treat during periods of high rainfall. In the study area, farmers reported treatments were given once a year if at all, though with no consensus on the best timing (Walker et al., 2015). Farmers could also use the information on seasonality to determine how often to check for anaemia in order to target animals with high burdens and high egg output.

Although the most efficient treatment timing was correlated with rainfall, a limitation of this model is that it depends on forward projection and therefore cannot directly inform a day to day decision on when to treat. Treating at the same time each year or based on the previous year's rainfall was suboptimal. A longer acting treatment was more effective than a shorter acting treatment (Figure S1 in Appendix S2) and may buffer some of the uncertainty around identifying the single optimal day for treatment. There are many potential scenarios for interactions between wild and domestic hosts; in this case, we assumed random mixing subject to overall density in each location. The simulations showed strong effects on the magnitude of transmission but rarely affected optimal treatment timing. Shared grazing with non‐competent domestic hosts is widely practised and can reduce the burden of worms in the target host (Mahieu & Aumont, 2009). As abundance of alternative hosts had a large predicted effect on transmission, it might be practical for managers to consider whether a particular set of alternative hosts is likely to amplify or reduce transmission.

Although migration has the potential to reduce disease prevalence (Johns & Shaw, 2016), the interaction between disease and migration may be different when there are multiple hosts. Under the migration scenarios examined, in which the migratory host had equal competence for the parasite as the stationary host, parasites moved across the landscape with migrating hosts, and 5%–18% of total worm burden in a given location was acquired from the other location. As there is potential for such movement, untreated wildlife may act as *refugia*, keeping susceptible parasites in the system (Van Wyk, 2001), or alternatively could move anthelmintic resistant parasites across the landscape and infect domestic hosts in new locations. Transmission of anthelmintic‐resistant *H. contortus* from wild to domestic ungulates has been demonstrated in the UK (Chintoan‐Uta, Morgan, Skuce, & Coles, 2014).

Validation of predicted risk by comparison with disease data was limited by a number of factors. Although predicted and observed seasonal patterns agree, *Q*_0_ explained only a small amount of the variation in FAMACHA score. In particular, the model output did not fit well to South African data (Table S1 in Appendix S2); however, these simulations did not include NDVI to scale seasonal grass growth, as NDVI was not available prior to 2003. Furthermore, sheep were treated during the study leading to suppression of cases over time. Previous work on this dataset has demonstrated the importance of variability in rainfall and temperature in driving FAMACHA score when individual‐level factors are accounted for (Babayani, Van Wyk, & Morgan, 2016).

The GLOWORM‐FL model on which the simulation was based is well parameterised using robust experimental data and validated against a direct measure of parasite development, *L*_3_ counted on pasture (Rose et al., 2015). Host and management factors, which are not accounted for in the model, could drive differences between the predicted development of *H. contortus* and observed cases of anaemia. Nevertheless, the extension to GLOWORM‐FL described in this study is a useful tool for broad simulation of the expected transmission patterns in new systems, and for optimising treatment, where longitudinal field data are not yet available. Such data are costly to collect, and the model can help to focus such efforts efficiently to calibrate to local conditions and test key predictions.

Despite the limitations of the model, the overall seasonal and spatial patterns do match those observed in the case study. Worm infection in the study farms was reportedly worse during and following the rainy season (Walker et al., 2015). Furthermore, higher parasite burdens were apparent in goats in the western, dry season range of the migratory hosts after, but not before, the rainy season (Table 4). After the rainy season, differences between the villages correspond to higher risk of parasites in areas with lower contact with wildlife. This pattern suggests a real and positive influence of migrating hosts, but is not seen in every year, and could be modified by climatic and other factors. Comparison of model output with more years of data would be beneficial, while well‐controlled treatment experiments could further elucidate causal patterns.

The assumption in the model that female worms produce 5,000 eggs per day results in high values of *Q*_0_ under prime conditions, such that one adult worm may give rise to 20,000 worms in the next generation in the absence of density‐dependent constraints. This is likely to be overestimated in the model due to the simplified assumption of how available *L*_3_ are consumed by hosts. There is high uncertainty around larval movement and availability on the complex herbage structure in the study area, while the model relies on estimates based on temperate grassland. Further experiments could elucidate how larval migration and subsequent consumption by potential hosts differs in the ecosystem found in the study area. Overall, unless there is substantial nonlinearity introduced by the difference between modelled and actual larval consumption, the modelled and actual values of *Q*_0_ should be proportional. This limitation will therefore not qualitatively change the seasonal patterns or model conclusions.

Using the current approach, we were able to quantify the potential impact of wildlife contact on parasite transmission. A similar method could be applied to other situations in which separation of host species is proposed as a method of disease control. In the case of helminths, the present study shows that amplification in transmission due to wildlife can be effectively managed by targeting treatment of domestic ruminants at peak transmission times. This is a tractable approach that can be applied to practical questions of disease and wildlife management in other mixed land use systems.

## AUTHORS’ CONTRIBUTIONS

J.G.W., E.R.M., K.E.E and J.A.vanW. conceived the idea and designed the methodology. J.G.W., H.R.V. and E.R.M. developed the model. J.G.W. collected the data and conducted the analysis. J.G.W. and E.R.M. led the writing of the manuscript. All authors contributed critically to the drafts and gave final approval for publication.

## DATA ACCESSIBILITY

Data available from the Dryad Digital Repository https://doi.org/10.5061/dryad.9jj84 (Walker, Evans, Rose Vineer, Van Wyk, & Morgan, 2017). Model code for data is also available on GitHub and Zenodo https://doi.org/10.5281/zenodo.1155649 (Walker, 2018).
