# Supplementary material for: Prediction and attenuation of seasonal spillover of parasites between wild and domestic ungulates in an arid mixed‐use system
Source: J Appl Ecol. 2018 Jan 28;55(4):1976–86. doi: 10.1111/1365-2664.13083 (PMC6032883; doi:10.1111/1365-2664.13083)
Supplement: Supplementary file 1 [file JPE-55-1976-s001.pdf]

Appendix S1: Supplementary Methods

Prediction and attenuation of seasonal spillover of parasites between wild and domestic ungulates in an arid mixed-use system

Josephine G. Walker[1,2,3,4,*], Kate Evans[1,3], Hannah Rose Vineer[2,5], Jan A. van Wyk[6], & Eric R. Morgan[2,7]

1. *School of Biological Sciences, University of Bristol, Life Sciences Building, 24 Tyndall Avenue, Bristol, BS8 1TQ, UK*
2. *Cabot Institute, University of Bristol, BS8 1UJ, UK*
3. *Elephants for Africa, Maun, Botswana*
4. *Population Health Sciences, Bristol Medical School, University of Bristol, Oakfield House, Oakfield Grove, Bristol, BS8 2BN, UK*
5. *School of Veterinary Sciences, University of Bristol, Langford House, Langford, North Somerset, BS40 5DU, UK*
6. *Department of Veterinary Tropical Diseases, Faculty of Veterinary Science, University of Pretoria, Private Bag X04, 0110, Onderstepoort, South Africa*
7. *Institute for Global Food Security, Queen's University Belfast, University Road, Belfast, BT7 1NN, UK*

*Correspondence author. `j.g.walker@bristol.ac.uk`

Supplementary Methods

Herbage density

Satellite-derived NDVI was used in the model to account for the change in grass biomass between the wet and dry season (Sheffield et al., 2014), and the sensitivity of the model to including grass biomass versus constant grass was explored. In the constant grass scenario, $\gamma = 1$, while in the alternative model NDVI, which is scaled from 0 to 1, is used for γ such that higher NDVI represents more grass, reducing the number of larvae consumed by diluting them. The amount of grass consumed (β) is therefore an additional scaling factor, which was assumed to be 1 for all host types. We assumed random mixing of hosts and of larvae on pasture.

Model validation

The ability of the model to predict timing of clinical cases was determined by comparing the predicted Q_0 with one host to the observed clinical cases of haemonchosis as measured by FAMACHA score (Van Wyk & Bath, 2002) in goats in the study area in 2013-2014 (Walker et al., 2015). The same validation exercise was completed on data from two farms in the summer rainfall region of South Africa, the first near Emalahleni from 1997-2000 (Babayani, 2016) and the second near Ermelo from 1999-2005 (Reynecke et al., 2011). We used a logistic linear mixed model with 'pale' (FAMACHA score 3 or greater) as the response variable and goat identifier as a random intercept to account for repeated observations of the same animal. We tested Q_0 as a fixed effect (scaled to mean 0 and S.D. 1) on the same date as each

observation and offset at weekly intervals with Q_0 values lagged for up to 8 weeks before the observation to allow for the time from egg deposition to clinical infection, and selected the best lag by AIC. These models were compared to a random intercept-only null model, as well as a model with expected rainy season (November-March) as a binary predictor, and scaled precipitation as a predictor. Predicted Q_0 was calculated with only one host at density 1 and establishment rate 0.5; the effect of grass growth scaling (NDVI) was included for the MPNP study area, but NDVI is not available for dates prior to 2003 so was not included for the South African data. Analyses were conducted using the R package `lme4` (Bates et al., 2015).

Simulation of anthelmintic impact

The impact of treatment was calculated for two anthelmintics that are widely used in Botswana: albendazole and albendazole–closantel combination. Albendazole has little residual effect and therefore egg output was suppressed for 14 days to represent the minimum time needed for newly ingested L_3 to develop into egg-laying adults (Anderson, 2000). Closantel provides a residual effect for approximately three weeks, so eggs were suppressed for 35 days to account for the residual effect plus development time. These time periods match the minimum timing for reapplication recommended by the drug manufacturers.

Migration scenarios

Migration was modelled as two alternative scenarios. The first was based on precipitation, whereby migratory hosts are in the wet season range if the

cumulative precipitation in the current or previous week was at least 2.5 mm (Bartlam-Brooks et al., 2013). The other scenario allowed for some animals to migrate earlier than others, and was defined by month, such that in December- February the hosts are in the east (wet season range), in August-October they are in the west (dry season range), and in the other months half of them are in each location (James Bradley, personal communication). Host density is assumed constant over time except as determined by migration, and when no migration is included, the full density of the hosts is assumed to be in both east and west, but there is no interaction between the two regions.

References

Anderson, R. C. (2000) *Nematode parasites of vertebrates: Their development and transmission.* 2nd edition, CABI Publishing, Wallingford, UK.

Babayani, N. D. (2016). Novel approaches to an automated decision support system for on-farm management of internal parasites of small ruminants. PhD thesis. University of Pretoria.

Bartlam-Brooks, H. L. A., Beck, P. S. A., Bohrer, G. & Harris, S. (2013) In search of greener pastures: Using satellite images to predict the effects of environmental change on zebra migration. *Journal of Geophysical Research: Biogeosciences*, **118**, 1427–1437.

Bates, D., Mächler, M., Bolker, B. & Walker, S. (2015) Fitting Linear Mixed-Effects Models Using lme4. *Journal of Statistical Software*, **67**, 1–48.

Reynecke, D. P., Van Wyk, J. A., Gummow, B., Dorny, P. & Boomker, J. (2011) Validation of the FAMACHA© eye colour chart using sens-

itivity/specificity analysis on two South African sheep farms. *Veterinary Parasitology*, **177**, 203–211.

Sheffield, J. et al. (2014) A Drought Monitoring and Forecasting System for Sub-Sahara African Water Resources and Food Security. *Bulletin of the American Meteorological Society*, **95**, 861–882.

Van Wyk, J. A. & Bath, G. F. (2002) The FAMACHA© system for managing haemonchosis in sheep and goats by clinically identifying individual animals for treatment. *Veterinary Research*, **33**, 509–529.

Walker, J. G., Ofithile, M., Tavolaro, F. M., Van Wyk, J. A., Evans, K. & Morgan, E. R. (2015) Mixed methods evaluation of targeted selective anthelmintic treatment by resource-poor smallholder goat farmers in Botswana. *Veterinary Parasitology*, **214**, 80–88.
