# Supplementary material for: Prediction and attenuation of seasonal spillover of parasites between wild and domestic ungulates in an arid mixed‐use system
Source: J Appl Ecol. 2018 Jan 28;55(4):1976–86. doi: 10.1111/1365-2664.13083 (PMC6032883; doi:10.1111/1365-2664.13083)
Supplement: Supplementary file 2 [file JPE-55-1976-s002.pdf]

Appendix S2: Supplementary Results, Tables S1-S3, and Figures S1-S3

Prediction and attenuation of seasonal spillover of parasites between wild and domestic ungulates in an arid mixed-use system

Josephine G. Walker[1,2,3,4,*], Kate Evans[1,3], Hannah Rose Vineer[2,5], Jan A. van Wyk[6], & Eric R. Morgan[2,7]

1. *School of Biological Sciences, University of Bristol, Life Sciences Building, 24 Tyndall Avenue, Bristol, BS8 1TQ, UK*
2. *Cabot Institute, University of Bristol, BS8 1UJ, UK*
3. *Elephants for Africa, Maun, Botswana*
4. *Population Health Sciences, Bristol Medical School, University of Bristol, Oakfield House, Oakfield Grove, Bristol, BS8 2BN, UK*
5. *School of Veterinary Sciences, University of Bristol, Langford House, Langford, North Somerset, BS40 5DU, UK*
6. *Department of Veterinary Tropical Diseases, Faculty of Veterinary Science, University of Pretoria, Private Bag X04, 0110, Onderstepoort, South Africa*
7. *Institute for Global Food Security, Queen's University Belfast, University Road, Belfast, BT7 1NN, UK*

*Correspondence author. `j.g.walker@bristol.ac.uk`

Supplementary Results

Migratory hosts and parasite movement

In the simulations, migratory hosts carried parasites acquired in one part of their range into the other (Fig. S3). Under a scenario with no migration, if a secondary host had the same density and establishment rate as the primary host, both hosts contributed in equal proportions to the total worm burden. When migration occurred, a proportion of the worms present in one location at a given time were acquired by migratory hosts in the other location (Table S3). Under the fixed-month migration scenario, there was a net movement of worms from east (wet season range) to west (dry season range). Under the precipitation-driven migration scenario, the pattern varied year to year (Fig. S3); in this scenario migratory hosts spent more time in the east (wet season range) than the west, contributing a large proportion of the overall burden on that side, while for both locations only about 7% of the burden came from the distant location. These results were generally robust to shorter (14 days) or longer (100 days) lifespans of the worm.

Supplementary Tables

Table S1 Percentage of farmers reporting shared water (W) or pasture (P) between wild and domestic ungulate species, by village (Questionnaire sample size). Only zebra, impala, wildebeest, buffalo, and elephants were provided as options on the survey, other species were suggested by interviewees.

		Gweta (8)		Khumaga (11)		Moreomaoto (11)		Phuduhudu (33)	
	Species	W	P	W	P	W	P	W	P
Cattle	Cape Buffalo	13	0	0	0	0	0	6	12
	African Elephant	63	100	91	82	55	55	30	58
	Impala	38	50	64	45	9	0	9	21
	Blue Wildebeest	63	75	82	82	18	0	36	61
	Plains Zebra	63	75	82	82	27	0	36	58
	Duiker	0	13	9	0	0	0	0	3
	Giraffe	0	0	0	0	0	0	3	9
	Greater Kudu	13	50	18	9	0	0	9	3
	Steenbok	0	13	9	0	0	0	0	0
	Warthog	0	13	0	0	0	0	0	0
Goats & Sheep	Cape Buffalo	13	0	0	0	0	0	9	18
	African Elephant	63	88	82	64	91	82	45	61
	Impala	38	50	64	45	36	0	15	36
	Blue Wildebeest	63	63	64	45	45	0	45	64
	Plains Zebra	63	75	73	55	55	9	48	73
	Duiker	0	13	9	9	0	9	0	3
	Giraffe	0	0	0	0	0	0	6	9
	Greater Kudu	13	50	27	9	0	0	12	6
	Steenbok	0	13	9	9	0	0	0	0
	Warthog	0	13	0	0	0	0	0	0
Horses & Donkeys	Cape Buffalo	13	13	0	0	0	0	6	6
	African Elephant	50	63	82	82	45	18	27	30
	Impala	25	25	64	27	18	0	9	18
	Blue Wildebeest	50	50	64	55	18	0	27	30
	Plains Zebra	50	50	73	55	18	0	27	30
	Duiker	0	0	9	0	0	0	0	0
	Giraffe	0	0	0	0	0	0	3	3
	Greater Kudu	0	25	27	9	0	0	6	6
	Steenbok	0	0	9	0	0	0	0	0
	Warthog	0	0	0	0	0	0	0	0

Table S2 Results of Logistic Mixed Effects models assessing correlation between observed clinical cases and predicted model output Q_0 including lags up to 8 weeks, rainy season, or precipitation, with animal ID as a random intercept. NDVI values were only included in the model for Makgadikgadi data as estimates of NDVI were not available prior to 2003. Effect estimate is per standard deviation in Q_0 due to scaling. Significance indicated by: * $P < 0.05$, ** $P < 0.01$, *** $P < 0.0001$.

Location	Model term	AIC	Estimate (Std. Error)	Wald z^2
Makgadikgadi (3570 observations of 1118 individuals)	null (random intercept)	3506.3		
	Q_0	3485.8	0.16516 (0.03501)	4.717***
	Q_0 lag 1	3491.2	0.15046 (0.03652)	4.12***
	Q_0 lag 2	3496.9	0.13437 (0.0398)	3.376***
	Q_0 lag 3	3501.4	0.11878 (0.04527)	2.624**
	Q_0 lag 4	3507.0	0.05531 (0.04832)	1.145
	Q_0 lag 5	3506.6	-0.06824 (0.05297)	0.198
	Q_0 lag 6	3490.1	-0.22601 (0.05420)	-4.17***
	Q_0 lag 7	3483.1	-0.24183 (0.04933)	-4.902***
	Q_0 lag 8	3476.5	-0.25344 (0.04595)	-5.516***
	Q_0 without NDVI	3489.5	0.14848 (0.03441)	4.315***
	season	3507.4	-0.10094 (0.10600)	-0.952
	precipitation	3506.3	-0.01549 (0.01122)	-1.38
Emalahleni (20053 observations of 939 individuals)	null (random intercept)	17977.8		
	Q_0	17943.0	-0.11800 (0.01960)	-6.02***
	Q_0 lag 1	17970.1	-0.06037 (0.01949)	-3.10***
	Q_0 lag 2	17979.7	0.00545 (0.019412)	0.28
	Q_0 lag 3	17966.3	0.06992 (0.01901)	3.68***
	Q_0 lag 4	17940.6	0.11894 (0.01897)	6.27***
	Q_0 lag 5	17882.5	0.18497 (0.01874)	9.87***
	Q_0 lag 6	17816.5	0.23851 (0.01869)	12.76***
	Q_0 lag 7	17764.6	0.27422 (0.01875)	14.62***
	Q_0 lag 8	17721.1	0.30023 (0.01874)	16.02***
	season	17642.7	0.73234 (0.04034)	18.15***
	precipitation	17933.0	0.018924 (0.002698)	7.01***
Ermelo (14605 observations of 1729 individuals)	null (random intercept)	6653.2		
	Q_0	6600.9	-0.25602 (0.03569)	-7.17***
	Q_0 lag 1	6619.0	-0.20715 (0.03511)	-5.90***
	Q_0 lag 2	6625.7	-0.18644 (0.03486)	-5.35***
	Q_0 lag 3	6641.8	-0.12447 (0.03427)	-3.63***
	Q_0 lag 4	6632.1	-0.16281 (0.03431)	-4.75***
	Q_0 lag 5	6633.2	-0.16690 (0.03595)	-4.64***
	Q_0 lag 6	6638.2	-0.15032 (0.03667)	-4.10***
	Q_0 lag 7	6655.0	-0.01342 (0.03643)	-0.37
	Q_0 lag 8	6653.7	0.04551 (0.03778)	1.2
	season	6633.5	0.40927 (0.09038)	4.53***
	precipitation	6653.8	0.003378 (0.002856)	0.237

Table S3 Proportion of total worm populations from local and distant region under migration scenarios. Scenarios include a primary stationary host, plus a second host which either does not migrate, or has fixed-month or precipitation-driven migration. Proportions of the total worm populations are presented for each of the two hosts in each simulation, with the burden of the migratory host divided by source location (local or distant to West or East reference indicated by columns). These results are shown for three lengths of worm lifespan (subcolumns).

Migration scenario	Host (Parasite Source)	Proportion of total burden (2003-2014)					
		West			East		
	worm lifespan (days)	55	14	100	55	14	100
Month	Stationary	0.663	0.691	0.638	0.673	0.646	0.702
	Migratory (Local)	0.176	0.175	0.174	0.222	0.245	0.195
	Migratory (Distant)	0.161	0.134	0.188	0.105	0.109	0.103
Precipitation	Stationary	0.657	0.661	0.655	0.735	0.734	0.736
	Migratory (Local)	0.273	0.282	0.269	0.186	0.199	0.179
	Migratory (Distant)	0.0696	0.0575	0.0765	0.0789	0.0673	0.0850
None	Stationary 1	0.5	0.5	0.5	0.5	0.5	0.5
	Stationary 2	0.5	0.5	0.5	0.5	0.5	0.5

Supplementary Figures

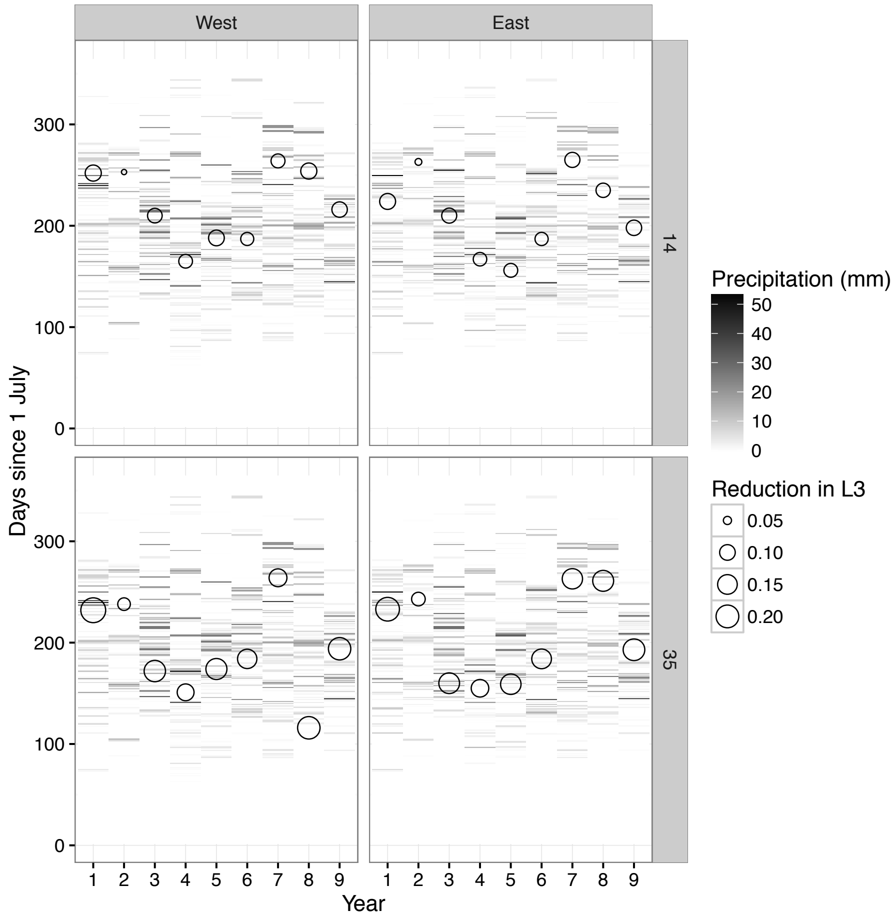

Fig. S1 Percent reduction in L_3 on herbage due to 14 day and 35 day treatments on west and east sides of the park in each simulation year (July 2003-June 2012), with years beginning 1 July (circles show optimal treatment date within the year, size scales with proportion of L_3 removed), and daily rainfall in mm (horizontal lines).

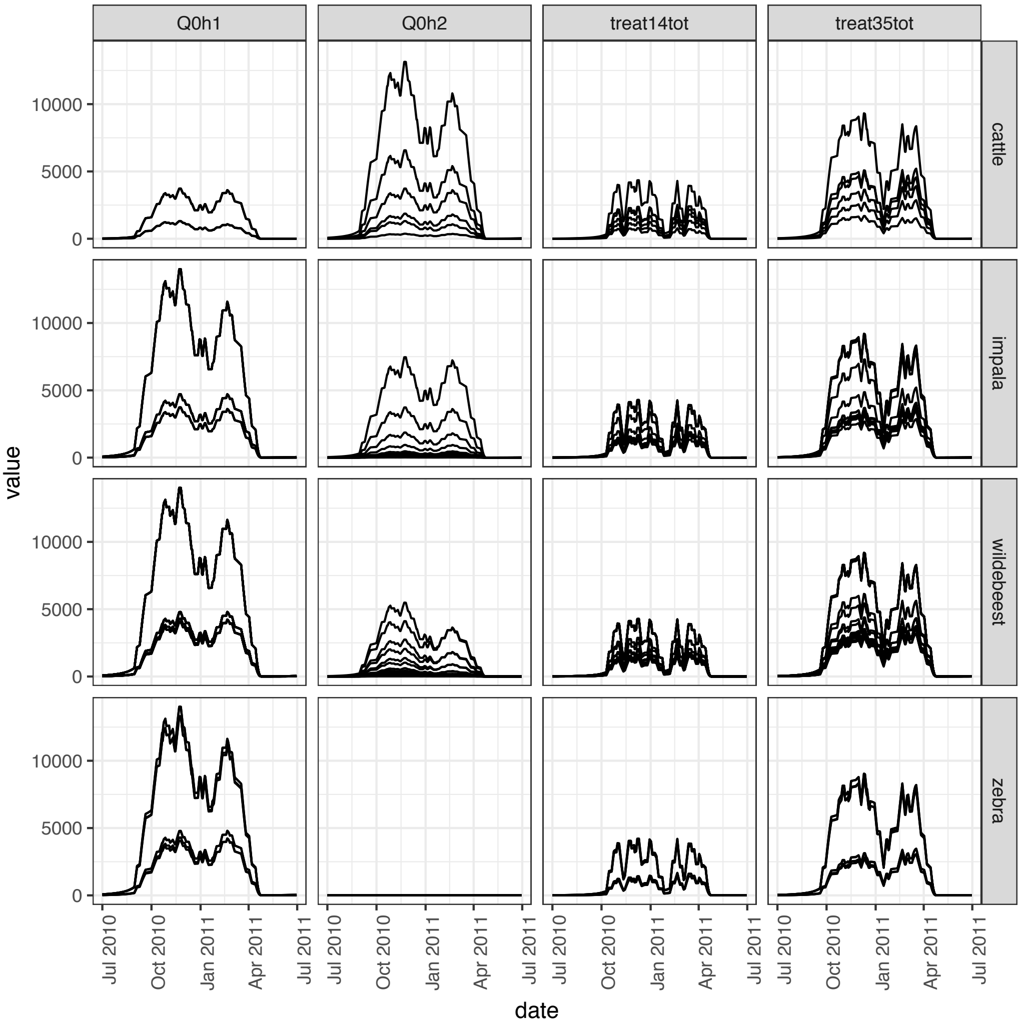

Fig. S2 Model output for realistic scenarios in the east, showing one representative year. Lines represent alternative parameterizations as presented in Table 2. Columns are model outputs, Q0h1 is Q_0 of the primary host, Q0h2 is Q_0 in the second host, treat14tot is the reduction in total Q_0 (Q0h1 + Q0h2) due to treatment on a given day, for treatment lasting 14 days and treat35tot is the same for treatment lasting 35 days.

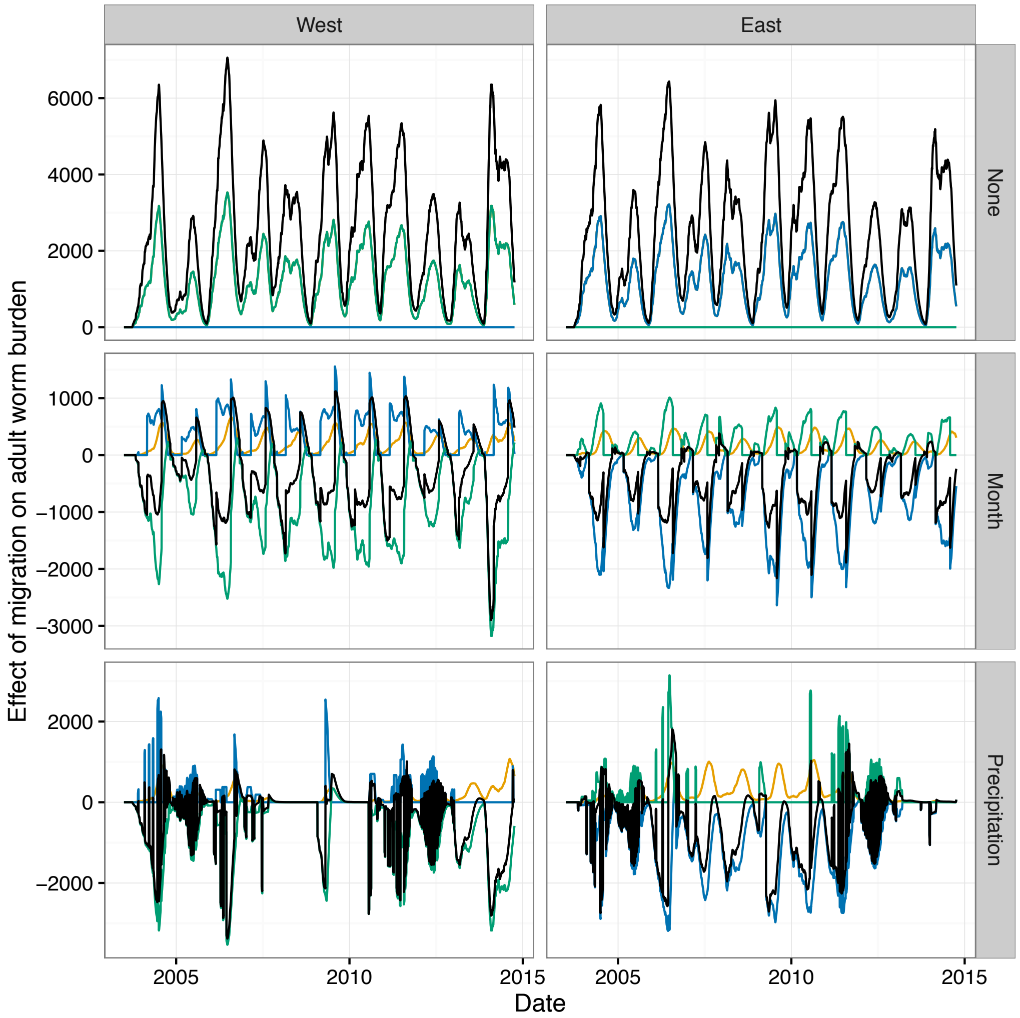

Fig. S3 Simulated adult worm burden by location (columns) and migration scenario (rows) over time, where establishment rate (ϵ) for both hosts is 0.5, density (ρ) for both hosts is 1, and lifespan of the adult worm (f) is 55 days. First row ("None") shows baseline scenario with no migration, while bottom two rows show the daily difference between burden for each host compared to no migration (top row) scenario. Black line, total burden in all hosts; gold line, burden in primary (non-migratory) host, blue line, burden in migratory host acquired in the east; green line, burden in migratory host acquired in the west. In the no migration scenario, the burden in the primary host is equal to the burden in the migratory host, therefore the gold line is not visible.
